# Barriers and Facilitators—Lessons Learned From a Randomised Trial to Implement Clinical Practice Guidelines for the Prevention of Coercion in Psychiatry

**DOI:** 10.1111/inm.70011

**Published:** 2025-02-14

**Authors:** Susanne Jaeger, Marie Kampmann, Johanna Baumgardt, Andreas Bechdolf, Felix Bühling‐Schindowski, Celline Cole, Erich Flammer, Julia Junghanss, Tilman Steinert, Lieselotte Mahler, Dorothea Sauter, Angelika Vandamme, Sophie Hirsch

**Affiliations:** ^1^ Ulm University Ulm Germany; ^2^ Centres for Psychiatry Suedwuerttemberg Ravensburg Germany; ^3^ Department of Psychiatry, Psychotherapy and Psychosomatic Medicine, Vivantes Hospital Am Urban, Vivantes Hospital Im Friedrichshain, Academic Hospital, Charité–University Medicine Berlin Berlin Germany; ^4^ Department of Psychiatry and Neurosciences at the Charité Universitätsmedizin, Campus Mitte Berlin Germany; ^5^ German Center for Mental Health (DZPG), Partner Site Potsdam Berlin Germany; ^6^ German Center for Mental Health (DZPG), Partner Site Mannheim‐Heidelberg Ulm Germany; ^7^ Department of Psychiatry and Psychotherapy, Clinics in the Theodor‐Wenzel‐Werk Berlin Germany

**Keywords:** aggression, coercion, de‐escalation, guidelines, implementation, qualitative

## Abstract

The PreVCo study (‘Prevention of Violence and Coercion’) investigated the effects of a structured programme for the implementation of guideline recommendations for the management of aggression and the prevention of violence and coercion in psychiatric hospitals in a multicentre randomised controlled trial with 55 participating wards. The intervention was a 1‐year individually tailored implementation programme supported by external consultants. An independent evaluation of the individual wards' process aimed at identifying barriers and facilitators in implementation. We conducted guideline‐based group interviews with 53 of 55 participating teams during the implementation process. The Consolidated Framework for Implementation Research (CFIR) was used for the qualitative analysis. Two coders independently coded the transcripts and conducted a summary content analysis. The focus was on facilitators and barriers in the implementation process. The design of the intervention, in particular the framework of a controlled study, external guidance and the opportunity to choose and adapt the implementation programme according to the wards' possibilities and needs, was generally perceived as useful and supportive. The context of pandemic management at the time of the study interfered with the implementation process, mostly because of organisational transformations, challenges for information exchange and increased workload of the staff. With regard to the wards participating in the study, the main facilitators were a receptive, collaborative ward culture, team spirit and previous experiences in successful transformation processes. Barriers included the demanding working situation, frequent fluctuation of staff and low team cohesion, obstacles in communication, a deficit‐oriented perception of patients and low priority of the implementation process. Provision of necessary resources by the organisation was not self‐evident. Stakeholders devoted to the ideas of transformation of psychiatry and reduction of coercion were important facilitators of the implementation; however, some employees kept a resigned attitude and could not be engaged. The analysis of barriers and facilitators shows that an implementation process of innovative routines on psychiatric wards can benefit from external, individually tailored guidance. However, the working conditions on psychiatric wards remain to be challenging.

**Trial Registration:** Clinical Trial Registration at www.isrctn.com with the identifier ISRCTN 71467851

## 1 Background

The use of coercive practices is one of the most crucial problems in inpatient psychiatry worldwide (Gill et al. 2024). To reduce coercion, a number of interventions have been shown to be effective in controlled trials (Hirsch and Steinert 2019). However, even for highly favoured individual interventions such as de‐escalation training and joint crisis plans, the evidence is still mixed (Du et al. 2017; Thornicroft et al. 2013; Radenbach et al. 2014). In recent years, several complex interventions, that is, specific combinations of individual measures have been developed, such as the Safewards model (Bowers 2014; Bowers et al. 2015; Fletcher et al. 2017; Baumgardt et al. 2020) or the Six Core Strategies (Huckshorn 2004; Putkonen et al. 2013; Wieman et al. 2014). Although these programmes to reduce coercion in psychiatry exist, evidence of their effectiveness in daily practice is inconsistent.

The PreVCo (‘Prevention of Violence and Coercion’) study was a multicentre randomised controlled trial (RCT) with 55 participating wards (Steinert et al. 2020; Steinert and Hirsch 2020). It investigated the effects of a structured programme for the implementation of guidelines' recommendations for the prevention of violence and coercion (DGPPN 2019). A total of 27 wards randomly assigned to the intervention group started the 1‐year implementation process in autumn 2020; the other wards in the wait list control group started 1 year later. As the guideline included a multitude of recommendations, and in reference to the six core strategy approach, the trial focused on a set of 12 measures recommended to be implemented on the wards in order to reduce coercion and violence (Steinert et al. 2020). These included, for example, regular team meetings to jointly evaluate recent coercive incidents and discuss alternative strategies; the use of a checklist for early detection of signs of tension in patients in order to prevent violent behaviour through individually applied measures; the involvement of peers as working colleagues on the ward; a mandatory debriefing with patients who had been subjected to coercive measures and the healthcare professionals involved. The participating wards were to select 3 of these 12 implementation recommendations they wanted to realise in the context of the study (alternatively: implementation of one complex intervention, e.g., Safewards). The primary outcome of the RCT was the reduction in the number of coercive measures. Coercive practices included seclusion, restraint and forced medication. One of the main findings of the RCT was a 43% reduction in the number of coercive measures on the intervention wards, 1 year after ongoing implementation activities. However, at the same time, the number of coercive measures also had decreased by 28% in the wait list control group. The difference in reduction between both groups failed to reach significance (Steinert et al. 2023).

Additionally, the degree of successful implementation was assessed, and the implementation process was monitored. The wards' progress was assessed by a 9‐point rating scale of the performance in each of the 12 implementation recommendations (Hirsch et al. 2023; Bechdolf et al. 2022) used at baseline and at the end of the study. After 12 months, intervention and wait list control groups differed significantly with a large effect size (Cohen's *d* = 1.48). The overall score increased significantly over time only in the intervention group (*p* < 0.001) (Steinert et al. 2023). This latter finding is in contrast to the results of a previous review that failed to show that guideline implementation has consistently positive effects on provider performance (Girlanda et al. 2017). To date, there are a large number of theories and empirical studies that can serve as a basis for the development of programmes to change clinical routines (e.g., Grol and Wensing 2004; Wensing et al. 2020; Lynch et al. 2018). In their review, Pereira et al. (2022) present the evidence of 30 strategies to promote guideline implementation aimed at healthcare organisations, healthcare providers and users. Tailored interventions seem to have an advantage to pure guideline dissemination strategies (Baker et al. 2015). The PreVCo implementation intervention was designed in line with the current knowledge base, for example, it used a multilevel approach, identified and involved relevant stakeholders, had a fixed time schedule, aimed at a precise definition of milestones to be reached within a certain period and employed regular feedback loops for evaluation of the progress. Integrative part of the intervention was the tailored, individual and flexible support provided by three experienced external implementation consultants. Their role was to provide theoretical and practical guidance and to enable the wards to successfully implement an individual selection of guideline recommendations. Each participating ward was given three workshops at different points in the course of the implementation process.

The aim of our qualitative study, which was conducted in parallel with the RCT, was to investigate the experiences of staff in implementing the PreVCo recommendations and to analyse the real‐life conditions that may have affected the success of the implementation. Thus, through interviews with the participating ward teams, we aimed to identify the factors and contexts that facilitated or hindered the implementation of the selected new practices. In particular, the fact that the intervention did not take place under laboratory conditions, but involved a living fabric of actors, structures and processes, which were also in constant interaction with factors outside the study, suggested a more detailed description and analysis.

The qualitative study, therefore, focussed on the circumstances surrounding the implementation process of the participating wards: Which factors favoured a successful implementation process? Which barriers could be identified? What role did the specific study focus on reducing coercive measures play? What lessons can be learnt for future guideline implementation efforts in general and in particular with regard to reducing coercion?

## 2 Methods

### 2.1 Study Design

We intended to capture the staffs' experiences of the implementation process on all participating wards in the RCT by means of guideline‐based group interviews that subsequently were to be analysed at the team level by qualitative content analysis to identify facilitators and challenges.

### 2.2 Sample

Overall, 55 wards located at 35 different study sites in Germany (both in urban and rural areas) participated in the RCT presented above. They all offered psychiatric treatment of adult inpatients with general mental disorders, substance use disorders or geriatric psychiatric disorders and had both involuntary and voluntary admissions. The median number of occupied beds per month was 18.7 (interquartile range IQR = 6.4), and the median number of admissions per month was 41.0 (IQR = 25.3) (for more information cf. Hirsch et al. 2023).

It was announced from the outset that in addition to the RCT, in which the study centres participated voluntarily, a qualitative accompanying study would also be conducted. For the qualitative analysis, we wanted to record the experiences of all 55 participating ward teams. As the teams were made up of employees from different professional groups, we planned to conduct group interviews with two to five team members representing different professional backgrounds. In favour of low‐threshold and voluntary participation, we left the selection of the individual interview participants to the wards themselves. We did not collect any socio‐demographic data from the individual interview participants, apart from their professional background and position in the company. The individual participants in the group interviews did not receive any incentives.

### 2.3 Data Collection

Each ward team was supposed to be interviewed at least once in the course of the implementation process. To cover all wards and all phases of the implementation process, the wards were randomly assigned to one of three time points: The interviews were to take place approximately 6–10 weeks after the first, second or third implementation workshop. In addition, a random sample of *n* = 10 wards was drawn from the wait list control group for a preimplementation interview. Similarly, a random sample of *n* = 10 wards was drawn from the intervention group for follow‐up interviews approximately 6–9 months after the last workshop. Thus, a total of 75 interviews were scheduled. The interviews were to be conducted optionally as a telephone or video conference, depending on the ward's capabilities.

**FIGURE 1:**
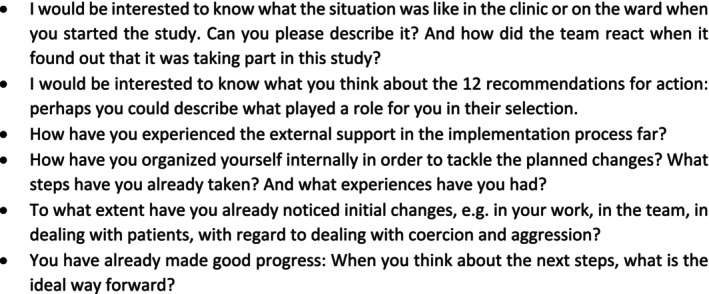
Exemplary questions of the interview guideline (after workshop 2).

We conducted semistructured problem‐centred interviews according to Witzel (2000) with only a few guiding questions and conversation as free as possible. The focus was on the participants' experiences with the implementation conditions, processes and effects (e.g., see Figure 1). The interview guidelines were developed according to the approach of Helfferich (2011). Since the interviews took place at different stages of the implementation process, the interview guidelines included overarching themes, but also, depending on the specific implementation stage, explored specific aspects in greater depth. To integrate possible phase‐specific experiences into the guidelines, we conducted focus groups with the implementation consultants in advance. The interviews were scheduled to last 30–45 min.

The implementation consultants informed the responsible key persons of the 55 wards about the upcoming interviews. We contacted the key persons by email and explained the background and procedure of the qualitative study using an enclosed information sheet. At the beginning of the interviews, the participants were informed in detail about the procedure and purpose of the study and had the opportunity to ask questions. Explicit reference was made to anonymity and confidentiality as well as to the independence of the qualitative research from the implementation coaching and the RCT. After having received informed consent, the number of participants, their professional background and the type of ward were assessed. The audio recording of the interview was then started.

The recordings were transcribed by a research assistant using a simplified transcription (Dresing et al. 2015). For data protection reasons, different speakers were generally only distinguished from each other by numbers (‘Person 1’). In a further step, the transcripts were pseudonymised: Wards were labelled with a code known only to the interviewers. Names and places were also pseudonymised or deleted.

The interviews and subsequent content analysis were conducted by two trained researchers (M.K. and S.J.) who were part of the PreVCo study group but were not involved in the RCT and its procedures. As the qualitative process evaluation took place at the same time as the implementation process, no interview content was shared with colleagues working on other parts of the PreVCo study until the end of the implementation phase to avoid influencing the RCT results.

### 2.4 Data Analysis

A summary content analysis according to Mayring (2014) was carried out in an alternating inductive and deductive procedure. The principle of openness prevailed. In addition to predefined categories, further categories could be added while the analysis progressed in order to cover relevant statements otherwise not sufficiently represented by the coding sheet. The entire interview was chosen as the level of analysis, with no distinction made between the individual speakers. The stage of the implementation was also not taken into account in this analysis.

**FIGURE 2:**
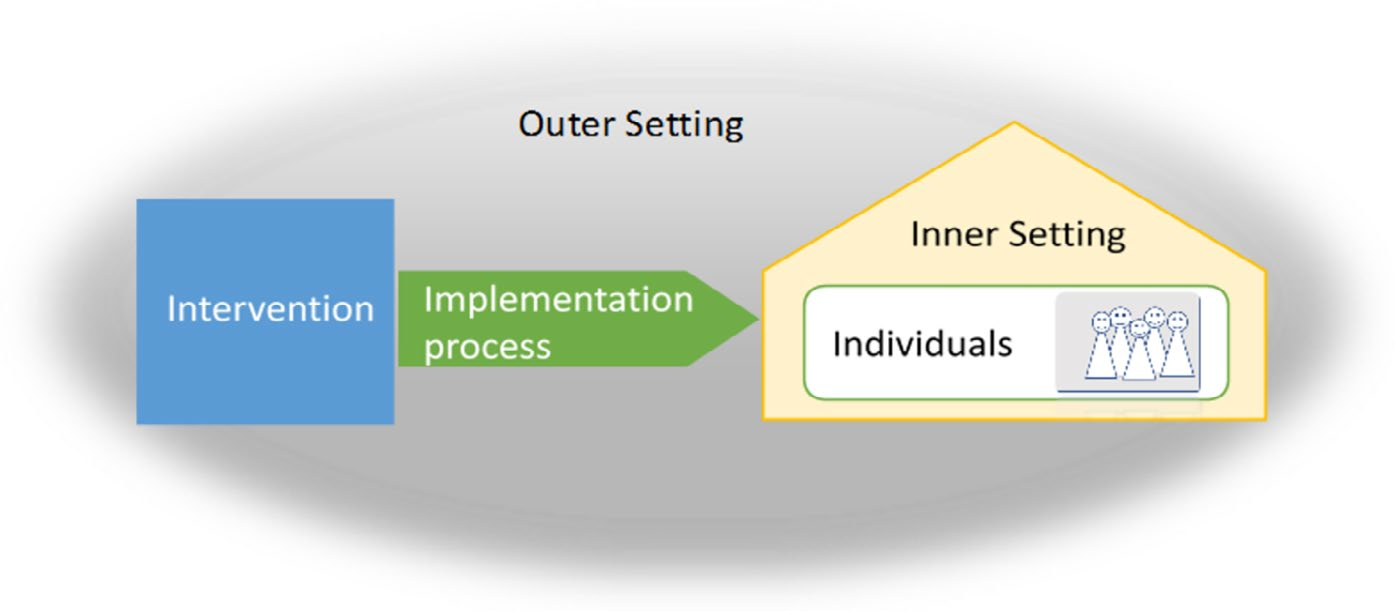
Main categories of the qualitative analysis of facilitators and barriers according to CFIR (figure adapted from Pinto et al. 2018).

Transcripts were coded in accordance with the Consolidated Framework for Implementation Research (CFIR; Damschroder et al. 2009) that provides relevant categories to identify and specify facilitating factors and barriers at five different domains: (1) the outer setting, (2) the intervention, (3) the inner setting, (4) the individuals involved in the implementation process and (5) the implementation process (see Figure 2). According to this framework, a successful implementation can take place if the conditions are favourable in all of these domains.

We defined the domains as follows:

- Outer setting: General and societal conditions in which the study and the implementation process took place including the perception of external events, legal requirements and societal values as well as networks and cooperation between the ward or staff members and other wards, clinics or organisations.
- Intervention: All procedures offered to the participating wards within the context of the PreVCo study including the option to participate in a study, the study design, the set of implementation recommendations the wards could chose to implement, the external support by trained consultants and the implementation workshops.
- Inner setting: Structures, processes, values and the current conditions on the participating wards in general, but also on specific supportive factors and barriers in everyday working conditions that were relevant for the study.
- Individuals' domain: Statements about the level of knowledge, motivations, values and attitudes of the employees of the participating ward.
- Implementation process: All steps taken by the wards to implement the guideline recommendations—from exploring the wards' needs and goals, through the selection of specific implementation recommendations and the involvement of relevant actors to the realisation of changes according to plan and activities to ensure the sustainable use of the implemented changes.

After an initial breakdown of the transcripts and assignment of statements to domains and categories, the statements were broken down again into the individual subcategories and summarised in terms of their potential to promote or hinder successful implementation of the recommendations.

MAXQDA 20 software was used for the qualitative content analysis.

### 2.5 Ethics

The primary ethical approval for the RCT and the accompanying qualitative study was obtained from the Ethical Committee of the Ulm University on September 4th, 2019, No. 55/19 and, subsequently, from the responsible ethical committees for the participating hospitals. Written informed consent for participation in the interviews was not required for this study in accordance with the national legislation and the institutional requirements.

## 3 Results

### 3.1 Sample Description

Seventy of the intended 75 interviews were conducted. In the intervention group (IG), all interviews could be realised as planned. In the wait list control group, it was not possible to conduct three preliminary interviews and two interviews after the second workshop, almost always due to lack of time or impossibility to get in contact. Overall, *n* = 53 of the 55 ward teams participated in the interviews at least once. Their treatment focus was predominantly in general psychiatry (*n* = 47), in five cases in addiction treatment and in one case in geriatric psychiatry.

A total of 157 employees took part in the 70 interviews, most of which were conducted with one person (24 interviews) or two people (23 interviews). In one interview, however, seven people were present. In 36 of the 46 interviews with two or more participants, the team members had different professions. The majority had a nursing background: A total of 84 nurses were involved in 57 interviews. A total of 38 doctors took part in 32 interviews. Other professions included psychologists, social workers and occupational therapists, as well as peer counsellors in two cases. Employees in management positions took part in 63 interviews, mostly with a nursing (50 interviews) or a medical background (25 interviews).

### 3.2 Facilitating Factors and Barriers to Implementation

The most frequently mentioned facilitators and barriers are summarised in Table 1.

**TABLE 1:** Summary of most frequently mentioned relevant facilitators and barriers to implementation.

	Facilitators	Barriers
Outer setting	External guidelines and legal frameworks Previous lack of networking across clinics combined with the wards' desire for an increase and learning from others	Covid‐19 pandemic and clinical pandemic management
Intervention	External source (conceptualisation, study design, coaching) Design of the intervention (format of a study, individualised, tailored support by external consultants) Individual choice and adaptability of the recommendations Expected relative advantage of implementation	Expected costs of the implementation (fear of imbalance of increased workload without added value) Changed format of the intervention: Online instead of face‐to‐face coaching workshops
Inner setting	Existence of good communication structures Team stability, team spirit and good cooperation within team Previous experiences in transformation processes and in reduction of coercion Team culture of common learning and development Awareness of patients' needs Awareness of need for change Accessibility of information and knowledge for any team member	Demanding working situation Lack of or high fluctuation of staff Low team cohesion Obstacles in communication and exchange Deficit‐oriented perception of patients—‘unavoidability of coercion’ Low priority of implementation process Low leader engagement Low provision of resources needed for implementation
Individuals	Conviction regarding the meaning and feasibility of changed practices Self‐confidence and self‐efficacy regarding transformation Individual qualification and experience with transformation processes	Lack of motivation for transformation within the individual team members Scepticism, hidden and open resistance to change Resigned attitude
Implementation process	Shared decision‐making in study participation, inclusion of the entire team in all processes, enabling multiprofessional workshop participation Elaboration of a team vision, thorough planning with clear distribution of tasks and fixed persons in charge and their substitutes in times of absence. Existence of ‘champions’ with a good standing within the team Implementation according to plan with regular reflection loops Regular evaluation of the implementation process within the team Standardised measures for evaluation	Lack of team participation in decision‐making processes (e.g., ‘imposed’ study participation) Workshop participation limited to selected team members Lack of multiprofessional commitment/absence of specific professions Absence of ‘champions’ Lack of elaboration in planning, unclear distribution of tasks, lack of fixed persons in charge and/or no clear substitution regulation Postponement/suspension of implementation steps Patients refusing to get involved or in a condition that challenged realisation (e.g., too agitated to participate in group discussions)

#### 3.2.1 Outer Setting

##### 3.2.1.1 Facilitators: Societal Framework and (Lack of) Cosmopolitanism

So I think it would be really great if we could have an exchange with others. That would be really great. So if you could do that more often.

There was widespread agreement with regard to external requirements, such as the implementation of legal requirements or working according to clinical guidelines. At the same time, a lack of ‘cosmopolitanism’ increased the desire to connect with others in some facilities. Damschroder et al. (2009, 7) define cosmopolitanism as ‘the degree to which an organization is networked with other external organizations. Organizations that support and promote external boundary‐spanning roles of their staff are more likely to implement new practices quickly’. In our study, some participants regretted the lack of cross‐departmental and cross‐organisational networking. They expressed a strong desire to connect with employees from other organisations and learn more about their practices. They welcomed the opportunity to benefit from the expertise of the implementation consultants and their experiences in other clinics. Thus, the limited networking to date combined with the desire to network with others across ward boundaries proved to be a motivating factor not only for participating in the study but also for implementing change on the ward.

##### 3.2.1.2 Barriers: The Pandemic and Its Management

We were always overcrowded and there were sometimes very very few employees on duty. of course that doesn't exactly make it easier to carry out such activities.

The Covid‐19 pandemic starting almost simultaneously with the start of the first implementation workshops was a fundamental and main external disruptive factor. It affected all domains of the implementation project: The intervention, the inner setting, the individuals and the process. Due to pandemic management, many hospitals reorganised their units and treatment processes. This affected the participating wards (e.g., changed distribution of tasks and work routines, changed patient allocation). The increased work strain in some staff members decreased the readiness for a change of routines. Many wards reported that managing the pandemic took top priority over all other changes. The originally planned implementation steps had often to be adapted to the pandemic‐related changing local conditions, structures and work priorities. Also, the intervention was affected: The coaching workshops had to be conducted online, not as planned face‐to‐face. This also implied that the wards had to organise the respective technical and spatial requirements. Workshop participants as well as consultants were confronted with unforeseen challenges.

#### 3.2.2 Intervention

##### 3.2.2.1 Facilitators: External Guidance and Study Design

And at the same time, there was something binding about knowing “She's coming back and we can't put our feet up now”.

Although the intervention had been developed by external actors and not by the ward itself, this was not perceived as interference, but rather as an opportunity. As a result of the structured guidance provided by external implementation consultants, the planning and implementation of changes were perceived as more binding. It was often welcomed that the consultants had experience in change processes and insights into the practice of other wards' approaches. They offered a different perspective on the established work routines.

The framework of a study with a defined endpoint and control group design, the guidance by external consultants, the continuous support through workshops and the adaptability of the procedures to the specific ward conditions (from the selection of implementation recommendations to the implementation schedule) were particularly positively emphasised in many interviews.

##### 3.2.2.2 Barriers: Fear of Negative Consequences Without Additional Benefit

Of course, there was also scepticism about how much work is involved, how much coercive measures are demonized and the risks involved in trying to reduce them—including for us.

Initially, isolated concerns were raised about interference by external parties without insight into local conditions but these voices disappeared during the course of the intervention. Since the concept implied that the wards themselves would decide on the recommendations they wanted to implement, they dispensed with the recommendations that they considered too complex. In particular, this referred to programmes that were likely to involve a great deal of bureaucracy or a multitude of different actors. It became apparent that employees feared additional work without additional benefits and reduced employee safety.

On some occasions, it was mentioned that the workshops—particularly due to the online format—were exhausting, too long and sometimes redundant in terms of content. In general, it was considered unfavourable that the workshops usually took place parallel to ongoing ward operations and that the staff members were not exempted from their work duties for participation.

#### 3.2.3 Inner Setting

##### 3.2.3.1 Facilitators: Ward Culture, Team Spirit and Experience in Transformation Processes

We had already implemented Safewards beforehand, so to speak, or were almost finished before PreVCo came along. So the team had experience. So it's actually quite an open team.

The ward cultures can be described as shared values and attitudes. In some wards, a shared interest emerged in challenging traditional practices, modernising psychiatry and its treatment concepts, learning about new methods and rethinking their own practices. The majority of teams recognised the need to change the current practices, not only for the benefit of users but also to create healthier working conditions for staff, which were often perceived as stressful. The respondents also expressed their desire to improve the quality of their work, for example, through better coordination of actions, more consistency and more transparency. Quite a few teams admitted that they were unhappy with professional traditions that they felt tended to escalate and were, therefore, interested in ways to change them.

In all teams, some pre‐existing communication structures were well connectable with specific implementation recommendations (e.g., monthly evaluation of coercive incidents found a fixed time slot in the regular team meeting). The multiprofessional team cohesion and collaboration (also cross‐ward), as well as a tradition of mutual collegial care and attention, were described to be supportive. Some wards described the advantage of a consistent, experienced team for coping with new tasks collaboratively, others emphasised the flexibility of a newly formed team or integration of new team members in order to overcome entrenched routines.

Experiences in successful implementation processes of new practices in order to reduce coercion boosted the team's confidence to manage further demands collaboratively. Many wards already employed established measures for dealing with aggression and promoting de‐escalation, some of them overlapping with the 12 innovations proposed in the context of the study (mostly de‐escalation trainings). It became clear that the selection was often also based on anticipated compatibility or connectivity with existing or previously tried practices.

Sensitivity to patients' needs and resources was also expressed in many interviews. In particular, this related to empathy with patients experiencing coercive measures or restrictions on autonomy. There was often a heightened awareness that some conditions on the ward triggered aggression in the patients, that is, confined spaces, deprivation of privacy, involuntary stay with other tense people. This strengthened the motivation to engage in ways of reducing coercion and to question routine practices.

##### 3.2.3.2 Barriers: Demanding Working Conditions, Shortness of Resources and Lack of Team Cohesion

And I'll just say that we're all fighting for survival. We are simply struggling with the fact that we have a clientele that is simply not adapted and does not want to adapt.

The most serious barriers to implementing the recommendations were found at the domain of the inner setting. Especially, high admission pressure, high user turnover and overcrowding were frequently cited as stress factors for the employees. Almost all wards reported a lack of sufficient, qualified and experienced staff or a high turnover of employees. Many part‐time employees and shift work made it difficult to work continuously towards a common goal. Multiprofessional, continuous collaboration was also hindered by different job‐specific work structures (e.g., shift work of nurses and job rotation of doctors). Extra efforts were necessary to pass on the information relevant to the study to all employees. This was exacerbated by measures taken in the wake of the Covid‐19 pandemic (e.g., contact restrictions). In addition to coping with the pandemic, some wards underwent further restructuring processes in parallel with study participation up to a fundamental reconceptualisation of the ward. This, too, was mostly perceived as an additional burden during ongoing routine operations, which pushed the implementation efforts into the background and slowed down study motivation. In everyday life, the focus was often primarily on survival and crisis management, not on reflection.

Some interviews revealed a sceptical attitude among the staff regarding the project and a deficit‐oriented view of the patients: Some patients were generally expected to be aggressive, and coercion was seen as unavoidable or indispensable for patients' and staff safety. By introducing new practices, there would even be a risk of becoming too permissive and jaded to overlook the subtle nuances of aggression and react too late. Thus, the need to change current routines was not recognised by all, and the decision of the ward managers to participate in the study was sometimes seen as a criticism of the wards' previous efforts to reduce coercion.

Certain recommendations proved to be incompatible with the ward organisation; for example, debriefing of coercive measures requires a minimum stay of the patients in one and the same ward and the simultaneous presence of certain staff members from different professional groups that had been involved. This may interfere with the internal organisation of the clinic, such as a policy of rapid transfer to other wards after diagnostic clarification, or with job‐specific work processes such as shift work, or with lack of time due to emergencies.

Leadership commitment was inconsistent across the different sites and ranged from high engagement in a few cases to a complete lack. The provision of specific resources for supporting the wards in the implementation process was reported only in isolated cases (personnel, financial resources, time and training measures). There were rarely extra benefits or resources by the clinic administration for the ward staff to participate in the workshops. The staff had in parallel to organise the ongoing ward operations.

#### 3.2.4 Individuals

##### 3.2.4.1 Facilitators: Motivation, Self‐Confidence and Experience

So it's actually the case that the majority of the whole team has caught fire and realized that this is something that brings something and is really beneficial—especially in terms of conflict.

More than half of the interviewed teams clearly expressed their conviction of the necessity and the effectiveness of implementing the recommendations. Scepticism among some team members in particular at the beginning of the process was frequently reported. However, motivation tended to increase with time and the implementation efforts were supported by a growing number of team members. Individual identification with the organisation strengthened the motivation to contribute to the success of the study. Many respondents were confident that they could cope well with the new tasks. Self‐efficacy was boosted in particular by previous successfully completed implementation processes, but also by positive previous experience with measures to reduce coercion. Specific professional qualifications (e.g., some of our interviewees were de‐escalation instructors) and training in the staff members were also beneficial.

##### 3.2.4.2 Barriers: Lack of Conviction and Motivation for Change

The employees have obviously not realized that they had the opportunity to change things that they want to change for the better.

Although many interviewees expressed conviction in the necessity and meaningfulness of the implementation, it was also clear that at the time of the interview, only about half of the teams were unanimously and clearly behind the change process. The problems ranged from individual staff members who were sceptical or resistant, to the entire team, making it a gruelling experience for those entrusted with the implementation. A wait‐and‐see, sceptical attitude in the staff members was overlaid with a certain degree of resignation, that is, the impression that hardly anything could be achieved in the existing service organisation, under these working conditions and with this type of patient.

#### 3.2.5 Implementation Process

##### 3.2.5.1 Facilitators: Shared Decision‐Making, Common Goals, Elaborate Planning and Regular Monitoring

You have to have the common goal in mind and you should know why you are actually doing something like this. A preliminary status quo survey like the one we had helps. You simply go through the points and see “Where do we stand? Are we satisfied or is there still room for improvement?”

Certain elements of the implementation project proved to be particularly beneficial: Shared decision‐making right from the beginning, the development of a shared team vision to decide on suitable measures to be implemented, elaborate planning with a timetable and milestones and regular evaluation and reflection on the ward's progress.

The development of a shared team vision at the beginning of the study provided a positive framework for the possible changes, with the opportunity to critically reflect on the own work situation, which increased motivation for change. Many interviewees particularly appreciated that they were given space and time to reflect on the practices applied to date. The moderation by external consultants in developing a plan for the ward was perceived as helpful. Clear definitions of common goals and milestones were important in the planning process, as was breaking down the measures into small steps so as not to overburden the wards. It was also necessary to have clearly named responsible persons and their deputies for each of the selected innovations.

The commitment of a critical mass of team members from all professions from the outset was crucial to the success of the implementation. To increase engagement, it seemed essential to enable participation in the implementation workshops for employees of all professions. In some teams, the significance of the project was emphasised by declaring the workshop a team day with obligatory attendance. The presence of ‘champions’ (employees committed to the change ideas) on the ward who enjoyed the trust of colleagues was favourable. The position of staff members who already advocated ideas of de‐escalation and noncoercion was further strengthened by study participation, and it was helpful when people in leadership positions were committed to the implementation to succeed.

Regular evaluation of the progress by the team seemed to be a crucial element in the implementation process. It reminded staff members of their visions and plans, added to keeping the ward in line, and in case of obstacles it helped to adjust the procedures. In this context, the use of specific assessment scales was valuable for recording and visualising progress and change.

##### 3.2.5.2 Barriers: Lack of Participation, Vague Planning, Postponement or Suspension of Actions

That we were conscripted. I would like to emphasize that once again. I thought it was a strange action. I said “no” three times and was not listened to.

In particular, a lack of team members' participation in decision‐making processes emerged as a major barrier to getting the staff committed to the change process. Study participation was perceived by some respondents as being ‘imposed’ by the hospital management. Even in later stages of the project, the lack of involvement of performers in decisions undermined commitment to successful implementation. When only a selection of individuals was able to participate in the workshops, or when specific professions were completely absent, extra efforts of communication were needed to get all staff members informed.

In accordance with the study concept, there was a lot of individual leeway for the realisation of the plan, both in terms of time and content. On the one hand, this was appreciated by the participants due to unforeseeable everyday requirements. On the other hand, this also could lead to postponements of the planned implementation steps. Vague planning, unclear distribution of tasks, unclear determination of responsible persons and their substitutes for setting the plans into practice and a failure to get support from the staff members also proved unfavourable. Due to the pandemic and day‐to‐day challenges, the implementation often did not go as planned. Some of the 12 recommendations explicitly focused on interaction with patients (e.g., debriefing on coercive measures, certain elements of Safewards (Bowers 2014)). In some cases, it was reported that the patients concerned refused to participate (e.g., in debriefing) or their condition impeded a realisation (e.g., too agitated to participate in common conferences).

Reflection and evaluation processes on the status of implementation were found to be very helpful, but due to a lack of time, they took place almost predominantly during the three workshops, not as part of the ward routine.

## 4 Discussion

The PreVCo study was an ambitious trial to investigate the effects of a complex intervention (implementation of a set of recommended transformations in clinical routine by the support of external consultants) on the reduction of coercion on psychiatric wards. The perspective of this qualitative evaluation was on the ward teams' experiences during the implementation process. What was helpful and what impeded the successful implementation? What can be learned for future implementation efforts?

The interviews showed that the implementation of the guideline's recommendations in clinical practice is generally perceived as challenging but feasible, provided that the adaptability to local conditions is given, the perceived advantages outweigh the feared disadvantages in the eyes of the team members and the implementation is guided by external coaches. In particular, the practical support provided by external consultants in structuring and supporting the implementation process was emphasised as helpful. The role of external facilitation in the implementation of complex interventions is already known in implementation research, but has only recently been increasingly investigated (Girard et al. 2024). It was a striking side result that the interviewees were very interested in receiving feedback on the performance of their team in comparison to others and in learning about the practices and experiences of other ward teams. Thus, the external consultants also served as a ‘window on the world’.

However, the framing conditions of the implementation process were challenging, in particular the organisational context. In their systematic meta‐review of 25 systematic reviews on the implementation of clinical practice guidelines, Correa et al. (2020) have pointed to comparable adverse conditions as could be found in our study, for example, difficulties with teamwork, lack of commitment among the staff, lack of administrative support of the institution, lack of resources and lack of information. Similar findings were provided in a review by Cheraghi et al. (2023) who assessed reasons for resistance to change in nursing: Besides personal and interpersonal factors, organisational factors such as lack of support and participation by the management, organisational values and adverse working conditions played a strong role.

The Covid‐19 pandemic and its management have had a massive impact on the implementation process. The pandemic is known to have increased psychological distress and work pressure among mental health workers (e.g., Kane et al. 2022; Muller et al. 2020). Understandably, coping with this emergency was the top priority at the time of our study, while the implementation process took a back seat. The focus on survival and crisis management jeopardised the willingness to try something new. In addition, the regulations hindered the teams' ability to meet and communicate, and providing relevant information about the changed procedures meant additional effort.

Commitment to the implementation process varied in the individual team members and across teams. The reluctance towards change might be a general problem in attempts to transform a system's established and routinely used practices. However, there is evidence that in particular seclusion and restraint in acute psychiatry are conflicted issues, and especially frontline workers such as nurses have to cope with contradictory role expectations which can cause additional distress. For example, in an Australian study with 44 nurses, the interviewees talked about their fears about the potential elimination of restrictive practices and saw themselves as being blamed for both, the use of these practices and the consequences should they be abandoned (Muir‐Cochrane et al. 2018). Accordingly, the programme of implementation coaching by external consultants in our study relied on gain framing: It began with the team jointly developing a vision of how the ward should evolve to deal constructively with aggression and coercion and make psychiatry a good place to work. The integration of the new practices into clinical routines should not mean a loss of practices to keep control and security, but rather a gain in confidence to act and in practices to prevent coercion and manage aggression in a different way. However, up to the endpoint of the study, not all employees in all teams could be convinced of the advantages to revise current practices but feared increased workload instead. In a recent study, work pressure and burnout affected considerably the acceptance of intended change in mental health nurses. Direct care staff perceived changes in general more negatively and felt more powerless and less confident than senior staff (Laker et al. 2019).

The ward climate in particular proved to be an important predictor of staff openness to change (Laker et al. 2020). In this context, it was certainly unfavourable that some teams felt that participation in the study was imposed by their organisation without being asked to decide for themselves or being provided with the necessary resources. According to the interviewees, the main problems, therefore, appear to be adverse working structures and employees who could not be convinced that their efforts could make a difference. Both aspects (feeling in control of the change process and trust in the usefulness of changed practices) are already emphasised in other implementation studies of clinical guidelines, for example, for depression by Forsner et al. (2010). It seems sensible to pay particular attention to these points when considering changes to current practices, and in particular in the context of implementing practices to reduce coercion. This was recently reaffirmed by a study conducted in the Netherlands on the implementation of high and intensity care: Again, the main barriers to change included lack of formal organisational support, resistance to change and scarce availability of resources (van Melle et al. 2021).

On the other hand, there was a strong interest in practitioners in how to improve aggression management and reduce coercive measures, in order to make inpatient psychiatry a better place for users and staff alike.

## 5 Limitations

Due to the priority of gathering experiences from all participating wards and from all phases of the implementation process, the course of implementation on the individual wards and the varying perspectives of different professions were not evaluated in depth. A summarising evaluation of single implementation recommendations was not possible, since the programme was individually tailored according to the ward teams' preferences. We decided to conduct this qualitative research independently of the intervention itself in order to avoid influencing the ongoing intervention process and the results of the study. However, as the two qualitative researchers were a (visible) part of the study team, they were not completely ‘independent’ or ‘external’ in the strict sense—neither in the eyes of the participating wards nor in terms of the contextual knowledge they had gained as members of the research team. The interviews were conducted with self‐selected participants, often employees in management positions which might have led to an underreporting of negative experiences. Selection effects, a reporting bias and loyalty effects or conflicts of interest of individual participants cannot be ruled out. The use of CFIR (Damschroder et al. 2009) as coding framework has led to meaningful results. However, there are some inconsistencies in the coding system that now have been improved in an updated version (Damschroder et al. 2022).

## 6 Conclusions

The interviewees' detailed descriptions of support factors and barriers enabled many aspects to be identified that should also be taken into account in future implementation projects. The implementation of guideline recommendations in everyday ward life, even in the context of the conflicted area of coercion reduction, is feasible if it is tailored to the individual conditions of the ward and if a critical mass of team members is behind the process. In particular, it is advantageous if this process is accompanied over a longer period of time by external experts who are experienced in team processes and are, therefore, credible and methodologically secure. Successful implementation requires time and resources for communication, joint reflection and review, which should be provided in advance and during the process. Team spirit and team cohesion play an important role. In order to strengthen and maintain employee motivation, it is important that all employees feel adequately supported by the organisation, are involved in the process and can participate in decision‐making. To strengthen motivation, it is also important that team members are able to recognise the benefits of the change, particularly for their own work in the ward, as can be achieved, for example, by developing a positive shared vision at the start of the process.

## 7 Relevance for Clinical Practice

The integration of recommendations from clinical treatment guidelines into daily clinical practice to reduce coercion can be facilitated through a structured, individually tailored implementation programme with long‐term guidance and support from external consultants. From the outset, working conditions, team processes and staff attitudes that may hinder the implementation process should be addressed and the necessary resources provided by the administration so as not to jeopardise staff commitment to change.

## Author Contributions

We confirm that all authors listed contributed to the manuscript according to the guidelines of the International Committee of Medical Journal Editors, and that all authors are in agreement with the manuscript.

## Ethics Statement

The primary ethical approval for the RCT and the accompanying qualitative study was obtained from the Ethical Committee of the Ulm University on September 4th, 2019, No. 55/19 and, subsequently, from the responsible ethical committees for the participating hospitals. Written informed consent for participation in the interviews was not required for this study in accordance with the national legislation and the institutional requirements.

## Conflicts of Interest

TS declared that he had received funding for other research projects from government bodies and for the development of the guideline from the German Association for Psychiatry and Psychotherapy (DGPPN). All other authors declared that they had no conflicts of interest.

## Data Availability

The authors have nothing to report.
